# Genital gender‐affirming surgery trends in Germany: Total population data with 19,600 cases from 2006 to 2022

**DOI:** 10.1111/andr.13762

**Published:** 2024-09-17

**Authors:** Cem Aksoy, Sascha Wellenbrock, Philipp Reimold, Philipp Karschuck, Mahmut Ozturk, Tobias Hirsch, Michael Sohn, Nicole Eisenmenger, Sabine Kliesch, Saskia Morgenstern, Aristeidis Zacharis, Johannes Huber, Luka Flegar

**Affiliations:** ^1^ Department of Urology Philipps‐University Marburg Marburg Germany; ^2^ Department of Plastic Surgery Center for Transgender Health University Hospital Münster Münster Germany; ^3^ Reimbursement Institute Hürth Germany; ^4^ Center for Reproductive Medicine and Andrology/Clinical and Surgical Andrology Center for Transgender Health University Hospital Münster Münster Germany; ^5^ Department of Urology AGAPLESION Markus‐Hospital Frankfurt Germany

**Keywords:** gender‐affirming surgery, health services research, population‐based study, transgender, treatment trends

## Abstract

**Purpose:**

To delineate the current trends regarding gender‐affirming surgeries (GAS) in Germany.

**Methods:**

Analysis of German hospital quality reports from 2006 to 2022 was conducted using the reimbursement.info tool. The German procedure classification (OPS) codes 5‐646.0 for masculinizing‐ and 5‐646.1 for feminizing surgery were assessed to identify GAS. Linear regression models were utilized for the analysis and depiction of current trends.

**Results:**

A total of 19,632 gender‐affirming procedures were performed during the study period with an exponential increase over the years. Masculinizing surgeries increased from 246 in 2006 to 1291 cases in 2022 (increase by 424%; *p* < 0.001). The highest annual increase of 37.2% in numbers was from 2018 to 2019 (from 1235 to 1694 cases). Feminizing surgeries increased from 180 cases in 2006 to 799 procedures in 2022 (increase by 343%; *p* < 0.001). The cases increased most between 2015 and 2016 from 277 to 502 cases (81.2%). The number of hospitals offering these surgeries expanded from 24 in 2006 to 29 in 2022 (21% increase; *p* < 0.001).

**Conclusion:**

This study demonstrates an exponential growth in numbers feminizing and masculinizing of GAS performed each year in Germany. Furthermore, a discernible trend emerges with a propensity for concentration of procedures within selected high‐caseload centers across Germany.

## 1 INTRODUCTION

Gender‐affirming surgery (GAS) aims to relieve gender dysphoria related to phenotypical gender incongruence. Apart from the psychological and hormonal treatment, GAS creates a concordance between the self‐identified gender, their physical appearance and functionality in daily life.^1^ This subject area and its treatment therefore encompass not only many medical specialties but also complex and interdisciplinary surgery.^2^ According to this, the GAS‐spectrum can be divided into “facial surgical procedures” (facial feminization/masculinization, vocal cord surgery, laryngeal chondroplasty), “top surgical procedures” (breast augmentation, mastectomy) as well as “bottom surgical procedures” (vaginectomy, hysterectomy, salpingo‐oophorectomy, orchiectomy, vaginoplasty, scrotal reconstruction, phalloplasty, metoidioplasty).^3^, ^4^, ^5^ A positive impact of GAS is well described in the literature and leads to improved quality of life (QoL) and reduced likelihood of the mental health treatment.^6^, ^7^, ^8^ Present guidelines for GAS follow the World Professional Association for Transgender Health (WPATH) Standard of Care version 8, offering recommendations for supporting patients through their transition process.^9^

Due to cultural and ethnical barriers, reliable epidemiologic numbers of patients with gender dysphoria worldwide are lacking. Population‐based data, primarily derived from European and North American institutions, indicate that reported proportions of transgender and diverse (TGD) populations range from 0.02% to 0.08%, marking a 10‐fold to a 100‐fold increase over the last decades.^10^ Contrary to these findings, the first census‐based data analysis in Canada 2021 accounted for 0.33% TGD of the population 15 years of age or older.^9^ The German Society for Transidentity and Intersexuality published a similar estimated number of 0.41%.^11^

In general, one can assume a noticeable increase in numbers of GAS worldwide.^12^, ^13^ Nevertheless, epidemiologic data obtained from solid sources depicting current trends and a status quo in Germany are lacking. Therefore, the objective of this study is to investigate the latest trends in GAS using a national total population analysis.

## 2 METHODS

### 2.1 German hospitals’ quality reports

GAS were analyzed with the reimbursement. INFO tool (Reimbursement Institute, Hürth, Germany) based on billing data from German hospitals’ quality reports. Since 2005, hospitals have been required by law to publish quality reports and the first version was available in 2006. The OPS code “5‐646.0” was used to analyze masculinizing surgery and the OPS code “5‐646.1” to analyze feminizing surgery. The relevant patient cohort was identified similar to our previously described method.^14^

The geographical maps were created by using the software “EasyMap 11.1 Standard Edition” (Lutum+Tappert DV‐Beratung GmbH, Bonn, Germany).

### 2.2 Statistical analysis

Data were presented by absolute and relative frequencies. For detection of trends over time, linear regression models were applied. A *p* < 0.05 was defined to indicate statistical significance. SPSS Version 29.0 (IBM Corp., Armonk, NY, USA) was used for statistical analysis.

### 2.3 Ethics statement

The data presented in this study were obtained in accordance with the World Health Association Declaration of Helsinki in its latest version. Since the data extracted from the databases were anonymized and de‐identified prior to release, an additional ethics statement was not required by the local ethics committee of Philipps University, Marburg.

## 3 RESULTS

**FIGURE 1:**
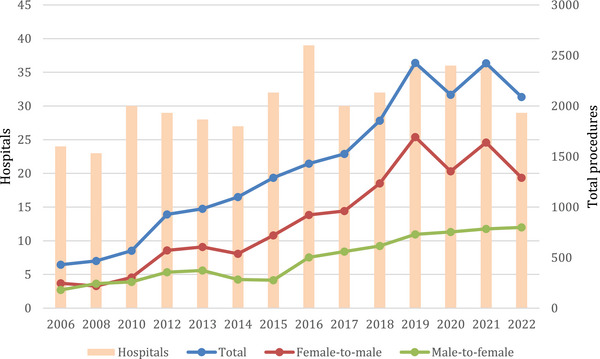
Yearly procedures and hospitals offering GAS from 2006 to 2021 (*Source*: Quality reports).

A total of 19,632 GAS procedures were analyzed. Figure 1 presents the annual number of procedures and hospitals providing GAS from 2006 to 2022. Masculinizing surgeries increased from 246 in 2006 to 1291 cases in 2022 (increase by 424%; *p* < 0.001). The highest annual increase of 37.2% was from 2018 to 2019 (from 1235 to 1694 cases). Feminizing surgeries increased from 180 cases in 2006 to 799 procedures in 2022 (increase by 343%; *p* < 0.001). The strongest increase was between 2015 and 2016 from 277 to 502 cases (81.2%).

**FIGURE 2:**
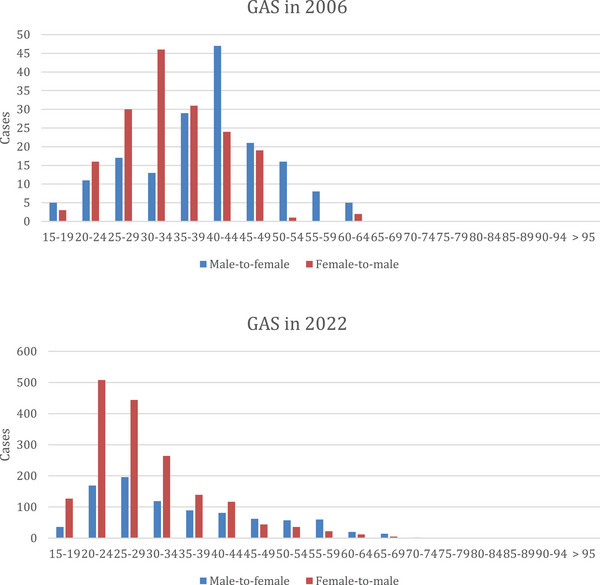
Age distribution for gender‐affirming procedures in 2006 and 2022 (*Source*: Quality reports).

Figure 2 shows the age distribution in 2006 and 2022. In 2006, 28.1% of patients who received masculinizing GAS were between 20 and 34 years old and 24.3% of patients who received feminizing GAS were between 20 and 34 years old. In the age group between 15‐ and 19‐years, cases increased in masculinizing procedures from 3 (1.2%) in 2006 to 127 (9.8%) in 2022. In 2022, 71.2% of patients who received masculinizing GAS were between 20 and 34 years old and 53.1% of patients who received feminizing procedures were between 20 and 34 years old. In the age group between 15‐ and 19‐years, cases increased in male‐to female GAS from 5 (2.7%) in 2006 to 36 (4.5%) in 2022.

**FIGURE 3:**
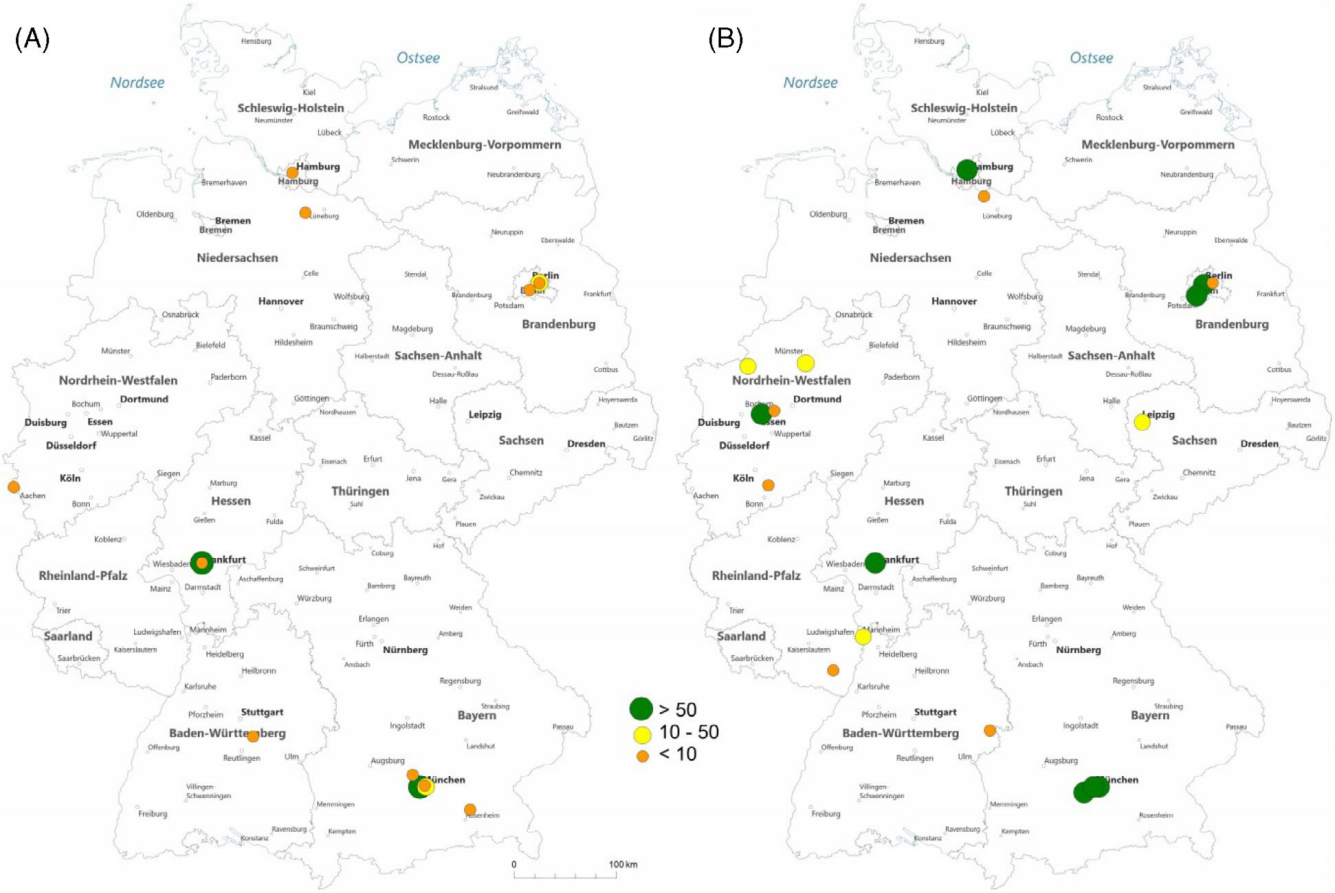
Female‐to‐male surgeries in 2006 (A) and 2022 (B) in Germany (*Source*: German hospitals’ quality reports corrected according to local case records for University Hospital Münster in 2022).

In 2006, 24 hospitals offered GAS which increased to 30 hospitals in 2022 (increase by 25%; *p* < 0.001). In 2006, 2 of 13 hospitals (15%) performed more than 50 masculinizing procedures per year, which increased to 9 of 18 (50%) in 2022 (Figure 3). In 2006, 72% of hospitals performed < 10 procedures per year and 13% of hospitals 10−50 procedures. In 2022, 33% of hospitals performed < 10 procedures per year and 17% of hospitals 10−50 procedures.

**FIGURE 4:**
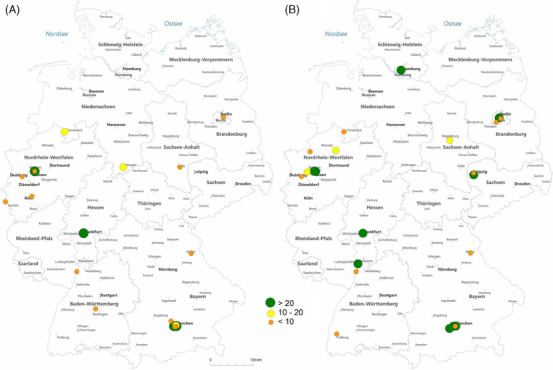
Male‐to‐female surgeries in 2006 (A) and 2022 (B) in Germany (*Source*: German hospitals’ quality reports corrected according to local case records for University Hospital Münster in 2022).

In 2006, 4 of 18 hospitals (22%) performed more than 20 feminizing surgeries per year, which increased to 8 of 23 (34%) in 2022 (Figure 4). In 2006, 63% of hospitals performed < 10 procedures per year and 15% of hospitals 10−20 procedures. In 2022, 60% of hospitals performed < 10 procedures per year and 6% of hospitals 10−20 procedures. In 2022, feminizing procedures were performed in 14 urologic departments (50%), 10 surgical departments (36%), and 4 gynecological department (14%). Five hospitals had a church‐based sponsor and six were university hospitals. In 2022, masculinizing procedures were performed in six urological departments (27%), nine surgical departments (41%), and seven gynecological departments (32%). Six hospitals had a church‐based sponsor and four were university hospitals.

## 4 DISCUSSION

The present epidemiological study analyzes nationwide demographic and structural data of transgender patients undergoing genital GAS in Germany in order to evince possible changes over the last two decades. To our best knowledge, this is the first total population study focusing on GAS in Europe. GAS in general, is becoming more and more popular due to increasing social acceptance driven by social media and the Internet.^15^, ^16^, ^17^ Moreover, GAS has been shown to improve QoL in gender dysphoric individuals.^8^, ^18^ Publications of surgical treatments in transgender individuals are sparse, despite an estimated prevalence of 25 million transgender individuals worldwide.^19^

### 4.1 Demographics

We have seen that the number of GAS multiplied. The total number of procedures increased almost 5‐fold between 2006 (*n* = 430) and 2022 (*n* = 2090). In the current literature, possible explanations mention the increased social acceptance of transgenderism in society, legislative changes as well as the increased access to health care providers as mainspring of this trend.^20^, ^21^, ^22^ The transition of the term “transsexualism” in the correct designation of “trans identity” or “gender dysphoria”, as we see, that is, in the adaption of the international classification of diseases (ICD 11), as from Diagnosis F64 in the field of “psychiatric diseases” to the field of “conditions related to sexual health”, is one important milestone of the afflicted outpacing their impairments and emphasize the importance of gender diversity.

In the observation period, the overall cases of masculinization operations (*n* = 12,306) were doubled compared to the amount of genital feminization operations (*n* = 6719).

These results are consistent with other studies resembling the upward trend in the United States during the last decades is frequently seen at our latitudes in Europe.^1^, ^22^

So far, most of the epidemiologic literature has been published by North American institutions.^23^, ^24^ Ross et al. presented a first report on annual numbers of GAS in the United States showing an increasing caseload of 20% between 2015 and 2016.^25^ But the German health care system differs strongly from the one in the USA.^26^ Canner et al. were able to reveal that among those, who were diagnosed with gender identity disorder (GID), only a number of approximately 10% were hospitalized for GAS.^1^

Interestingly, there was a high number of masculinization surgeries in our cohort with almost double the number of cases compared to feminization procedures. Ha et al. stated that out of all procedures the phalloplasty was the one less frequently asked for.^22^ One can argue that the steps of phalloplasty are in most cases up to five operations, while the vaginoplasty can be completed in only a single or two procedures. The pure microsurgical free flap is the generally customary term of the “phalloplasty” with the fear of total or partial flap loss subsequently leading to misinterpretation of the female‐to‐male genital affirmation procedures in general.

### 4.2 Age

Our analysis shows that patients are younger in recent years when they are undergoing genital GAS (2006 28% in the group of 20–34 years in the masculinizing sample; in 2022 71%; 2006 24% in the group of 20–34 years feminizing procedures; in 2022 53%). Our results further highlighted, that around 70% of those who underwent masculinizing interventions fell within the age range between 20 and 34 years, while approximately 54% of patients who opted for feminizing GAS were in the same age group. Similar results were presented by Wright et al. in a recently published cohort study from the US, including data from 2016 to 2020 in the Nationwide Ambulatory Surgery Sample and the National Inpatient Sample.^27^ The authors reported on a total of 48,019 patients who underwent GAS, including 25,099 (52.3%) who were aged 19–30 years.

Moreover, our study highlighted an increase of all masculinization operations in the age group between 15 and 19 years from 1.2% in 2006 to 9.8% in 2022. The same trend we also observed with feminization procedures in the age group between 15 and 19 years with 2.7% in 2006 increasing to 4.5% in 2022.

One important role, previously mentioned, is the impact of social media and online information, enabling young transident patients to better inform themselves at a younger age.^16^

### 4.3 Hospital analysis

In our study, we detected a substantial increase of case numbers as well as the number of hospitals offering the therapy. In 2022, a total of 29 German hospitals performed 2090 GAS operations. In 2016, we had up to 43 hospitals offering transgender patients these special treatments, but after this year the number declined. One can assume that implementing a facility of transgender operations in a multidisciplinary health care team approach consumes a large number of personal and financial resources which not every institution can afford. This team may include a primary care provider, plastic surgeon, urological surgeon or gynecologist, psychologist, as well as endocrinologist and a social worker.^28^

Another important factor is that genital GAS, especially masculinization procedures, are multiple‐staged procedures with long operation times and high complication rates, that is, phalloplasty up to 32%−54% or in genital feminization operation from 4% to 25%.^16^, ^17^, ^29^, ^30^, ^31^, ^32^ In this context, our study revealed an increase of high‐volume centers performing more than 50 procedures a year from 15% in 2006 to 50% in 2022. In the same time period, the number of low‐volume centers decreased from 72% in 2006 to 33% in 2022.

Our results further showed that urological, surgical, and gynecological departments were mainly performing GAS in Germany. While feminizing procedures were performed in 14 urologic departments (70%) in 2021, female‐to‐male procedures were performed in six urological departments (21%), respectively. Further, GAS was not only performed at university hospitals but also at church‐based sponsored hospitals. A survey among reconstructive urologists from 2020 showed that younger physician age and working for an organization interested in developing a multidisciplinary GAS program influence positive attitudes toward GAS.^33^ The WPATH administered their standard of care in their current guidelines for GAS, version 8, providing this multidisciplinary approach.^9^

The increasing number of hospitals performing high caseload increased from 14% to 30% for feminizing GAS as well as from 21% to 40% for masculinizing GAS suggesting this assumption. Nevertheless, the existing literature lacks granularity what is strictly insurance covered and what belongs to the ‘self‐paid’ operations although a nationwide S3‐guidelines for gender dysphoria already exists.^34^

### 4.4 Geographical allocation

Upon reviewing the maps we generated, it became evident that in 2022, in all major cities such as Berlin, Hamburg, Munich, Frankfurt, and the metropole region Rhein‐Ruhr, there was accessibility to transgender procedures for patients. Hence, GAS was offered all over Germany.

### 4.5 COVID‐19

Interestingly, measurements in 2020 and 2021 were set in a time, where there was a tremendous impact of COVID‐19 disease on the elective operation numbers all over the world as well as in Germany in 2021.^35^, ^36^ We observed a slight decline in case numbers in 2020 which are most likely attributed to the COVID‐19 pandemic. However, in 2021 especially masculinizing interventions increased again. Our working group recently reported on the implantation rates of testicular protheses in Germany. Similar, we also noticed a decrease in caseload in 2020, while transgender surgeries were the main driver for increasing numbers overall.^37^

### 4.6 Limitations

While providing a large retrospective analysis of GAS in Germany as well as analyzing current trends several limitations have to be addressed. The quality reports lack clinical data, which would greatly enrich the depth of our analysis. The quality reports may be also subject to documentation errors since they are prepared by the hospitals during the routine care. Due to insurance coverage and the complexity of GAS, our personal experience is that less than 5% of these special surgical procedures are performed in an outpatient setting. Furthermore, the applied operation‐codes “5‐646.0” for masculinizing‐ and “5‐646.1” for feminizing transformation procedure are not included in the German OPS‐outpatient catalog and could therefore strictly be used during the inpatient surgical treatment.^38^ The manifold GAS, masculinizing and feminizing, should not tempt observers to conclude numbers of patient data itself. One can only extrapolate on patient numbers to the factor 1/4−1/5 in masculinizing procedures and 1/2−1/3 in feminizing, especially taken into consideration the multiple staged procedures on one hand (five in Phalloplasty's and mainly two Vaginoplasties), and the relatively high reoperation rate as mentioned earlier.

However, by accepting those limitations we were able to provide the first population‐based data on GAS in Germany over an extensive study period.

## 5 CONCLUSIONS

This study discloses a first demographic overview in the field of transgender health operations and depicts a significant increase of genital GAS performed in recent years in Germany. Moreover, we noticed a trend with a propensity for concentration of procedures within selected high‐caseload centers across Germany. The consistent increasing numbers, the growing number of health care providers, and the trend toward younger patients reveal a growing social acceptance, the legislative changes, such as the implemented self‐determination law, and overall adaption of transgender diversity to western societies. Regarding this trend of GAS, it is important to continue improving social acceptance and raising awareness of GAS in the society.

Further studies need to be performed to analyze for specific GAS procedures such as facial‐, top‐, and bottom transgender surgeries. In addition, follow‐up studies on GAS should be carried out with focus on quality of life as well as quality control of the surgical outcome.

## AUTHOR CONTRIBUTIONS

All authors whose names appear on the submission have contributed sufficiently to the scientific work and therefore share collective responsibility and accountability for the results. *Study concept and design*: Cem Aksoy and Luka Flegar. *Acquisition of data*: Cem Aksoy, Sascha Wellenbrock, Philipp Reimold, and Luka Flegar. *Analysis and interpretation of data*: Cem Aksoy, Sascha Wellenbrock, and Luka Flegar. *Drafting of the manuscript*: Cem Aksoy, Sascha Wellenbrock, Philipp Karschuk, Aristeidis Zacharis, and Luka Flegar. *Critical revision of the manuscript for important intellectual content*: all authors. *Supervision*: Luka Flegar and Johannes Huber.

## CONFLICT OF INTEREST STATEMENT

Dr. Cem Aksoy is founder and a member of PATE e.V. (Prevention and Advocacy of Testicular Education www.pate‐hodenkrebs.de). PATE is a non‐profit association that promotes education, prevention, and self‐help for testicular tumors and other diseases of young men. Nicole Eisenmenger is founder and director of RI Innovation GmbH. Dr. Johannes Huber reports grants and non‐financial support from Intuitive Surgical, Takeda, Janssen, Apogepha, Bayer, and Coloplast outside the submitted work. Mrs. Dr. Luka Flegar is a consultant for BK medical and received support and grants from Novartis and Astellas. All other authors declare that there is no conflict of interest.

## Data Availability

All datasets used in this work are stored centrally at the specific institutes (German Federal Statistical Office—Destatis; German National Centre for Cancer Registry data at the Robert Koch Institute).
